# Factor structure of the convergence insufficiency symptom survey questionnaire

**DOI:** 10.1371/journal.pone.0229511

**Published:** 2020-02-24

**Authors:** Amélia Fernandes Nunes, Pedro Lourenço Monteiro, António Santos Nunes

**Affiliations:** 1 Universidade da Beira Interior, Covilhã, Portugal; 2 Health Sciences Research Centre (CICS-UBI), UBIMedical, Covilhã, Portugal; 3 Research Unit in Business Sciences (NECE), Covilhã, Portugal; Faculty of Medicine, Cairo University, EGYPT

## Abstract

The purpose of this study is to analyze the factorial structure of the Convergence Insufficiency Symptom Survey questionnaire with 15 items, in order to identify latent dimensions that can contribute to a more focused implementation. The questionnaire was self-administered in paper by 183 university students, in the age span of 18 to 30. Both Kaiser-Meyer-Olkin measure and Bartlett's sphericity test were performed to ensure the validity of the factorization. In order to analyze the principal components, factors which obtained eigenvalues greater than 1 were chosen. The extraction of factors was performed after computing a Promax rotation and a Kaiser criterion. In each extracted factor, the internal consistency was used to prove its reliability. Bartlett's sphericity test was statistically significant (p <0.001), and the Both Kaiser-Meyer-Olkin test was 0.89, confirming the factorization of this instrument. Exploratory factor analysis followed by a Promax rotation and scree plots graphic, extracted three factors that explained 62.1% of the total variance. The composition of each factor suggests the following meanings: Factor 1 (somatic sensation) includes 8 of 15 items; Factor 2 (impaired vision) includes 3 of 15 items; Factor 3 (cognitive performance) includes 4 of 15 items. Cronbach's alpha coefficient demonstrated good internal consistency (α> 0.75) in three dimensions. The multivariate statistical analysis of the Convergence Insufficiency Symptom Survey revealed a three-factor structure, so new forms of questionnaire analysis can be designed in order to obtain gains in the management of a symptomatic patient, using 3 subscores, one for each factor, instead of a total score. The factorial structure of the questionnaire can be used with a high level of confidence in future investigations, which aim to assess which dimensions are most affected in each type of visual changes and develop more targeted therapeutic performances.

## Introduction

Reading and other near activities are visually demanding tasks that can become difficult and uncomfortable with the manifestation of symptoms such as eye fatigue, text movement, blurring, and loss of concentration, even when visual acuity is good and with normal binocular vision [1–4]. Students who spend most of their time reading and writing tasks, represent a population highly predisposed to report visual discomfort associated with performing tasks at near distances [2,3,5]. The presence of these symptoms potentiates a decrease in visual performance and affects school performance [4,6], contributing to a decrease in quality of life [3]. The increase in the use of digital devices, has contributed to the increase of this type of complaints, which is considered a public health problem [3,7].

The visual discomfort can be caused by several factors, specially (1) eye problems, such as the presence of uncorrected refractive errors, accommodative or binocular anomalies [8,9] and (2) others situations, like unsuitable working conditions related to lighting and temperature levels, high continuous working time, short working distances, excessive use of electronic devices and mental status [2,3,7,10,11].

The attempt to evaluate both, the presence and intensity of symptoms, and identify a specific cause are fundamental aspects to define the best professional performances and are therefore challenges presented by others authors [12,13]. Complaints of visual discomfort cover a set of symptoms, usually called asthenopia or eye fatigue. Traditionally this set of symptoms are classified into two groups: refractive asthenopia and muscular asthenopia [14]. Sheedy also categorized complaints of asthenopia in two types: external symptoms and internal symptoms [15].

A number of questionnaires of visual discomfort, duly validated, are available to measure this parameter, of which the most frequently mentioned in clinical research are the questionnaire developed by Conlon and the Convergence Insufficiency Symptom Survey questionnaire (CISS) [5,16,17].

The CISS questionnaire, is presented as a reproducible instrument, with validity, with high internal consistency and capable of responding to clinical changes during the treatment of convergence insufficiency. The same questionnaire has also revealed added value in other situations like in quantifying symptoms of visual discomfort in students [18], in the signaling of subjects more prone to visual complaints, associated with the visualization of 3D screens [19], or evaluating the effects of wearing yellow spectacles on visual symptoms [20]. It has also been applied for evaluating symptoms severity in accommodative insufficiency [21]. However, it is an instrument that has been used as a tool with a single score and this may be one of the reasons why it has been pointed out as a tool with poor specificity for convergence insufficiency [4,11,22].

The aim of this study is to verify if the Portuguese version of the CISS questionnaire is factorable and, if so, to analyze its factorial structure, in order to identify the latent dimensions that may contribute to a more specific application of this questionnaire in clinical routine.

## Materials and methods

### Instrument

The instrument used for factorization was the CISS questionnaire, which is composed by 15 items. The response to each item ranges from "never" to "always" on a 5-level Likert scale. For data collection, the Portuguese version of the questionnaire was used [23].

### Participants

In order to make the suitable factorization of a given instrument, there are some directives regarding the potential sample size, and it is recommended to use 5 to 20 cases per variable, at least 10:1, and at best 200 subjects [24–26]. A total of 183 higher education students, aged between 18 and 30 years (mean: 21.4± 2.5), 51.5% of female respondents. Exclusion factors were strabismus and ocular surgery. All participants gave their written consent to the completion of the questionnaire and followed the guidelines of the Helsinki Declaration. The study was approved by the Ethics Committee of the Faculty of Health Sciences, University of Beira Interior (process CE-FCS-2012-27).

### Statistical analysis

**Fig 1:**
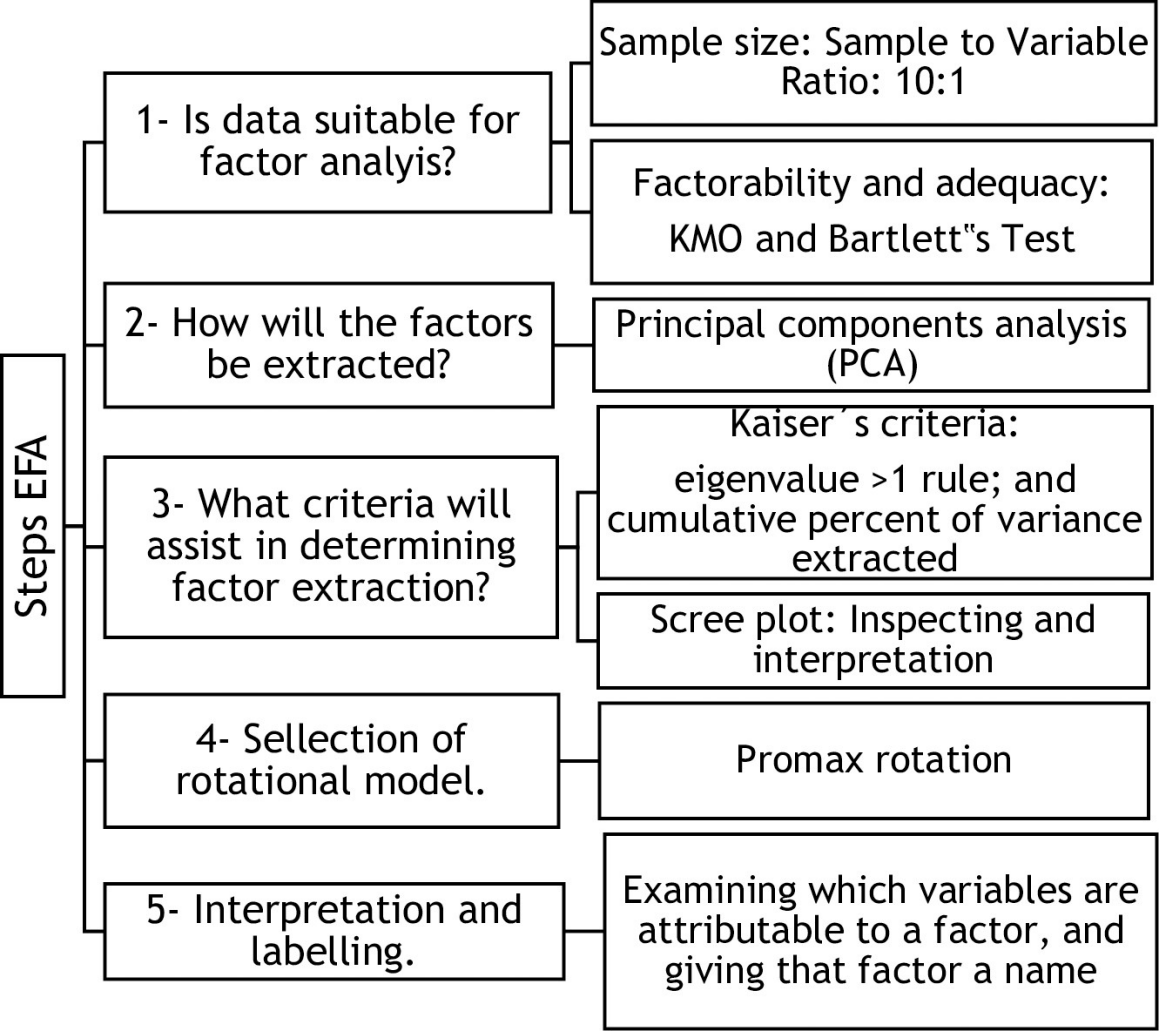
Steps followed in Exploratory Factor Analysis.

For statistical treatment of data, we used version 25 of the IBM Statistical Package for the Social Sciences (SPSS). Fig 1 shows the steps that were followed.

The Kaiser-Meyer-Olkin index (KMO) and the Bartlett Sphericity test were used to confirm the validation of the factorization of the variables. The KMO index, also known as sample adequacy index, is a statistical test that suggests the proportion of the item variance that can be explained by a latent variable [25]. Such an index indicates how adequate the Exploratory Factor Analysis (EFA) application is to the dataset. For interpretation of the KMO index, values less than 0.5 are considered unacceptable, values between 0.5 and 0.7 are considered reasonable; values between 0.7 and 0.8 are considered good and values greater than 0.8 are considered excellent. The Bartlett Sphericity test evaluates to what extent the (co)variance matrix is similar to a matrix-identity, that is, they do not correlate with each other. Bartlett's Sphericity test values with significance levels p <0.05 indicate that the matrix is factorable [24].

For the analysis of the main components we chose the factors that obtained eigenvalues greater than 1 in agreement with the Scree Plot. A percentage greater than 50% of the retained variance was accepted in the extracted latent variables, also referred to factors or dimensions [24]. In order to maximize the factorial weights of the high items and, at the same time to minimize the lows, the extraction of the main factors was performed after Promax rotation [25,26].

For each factor extracted, the internal consistency was also analyzed to test its reliability, using the Cronbach’s alpha. This coefficient expresses the consistency in the answers to a questionnaire, in order to conclude that they all measure the same parameter and that all can be added in a single score. Values of α greater than 0.7 are considered acceptable [26,27].

## Results

To begin the exploratory factorial analysis, an initial inspection of the correlation matrix was performed and the possibilities of reducing variables were verified. The data matrix was considered adequate since KMO was excellent (0.89) and the Bartlett's Sphericity Test (p <0.001) indicated that the correlation matrix is not an identity matrix.

**Fig 2:**
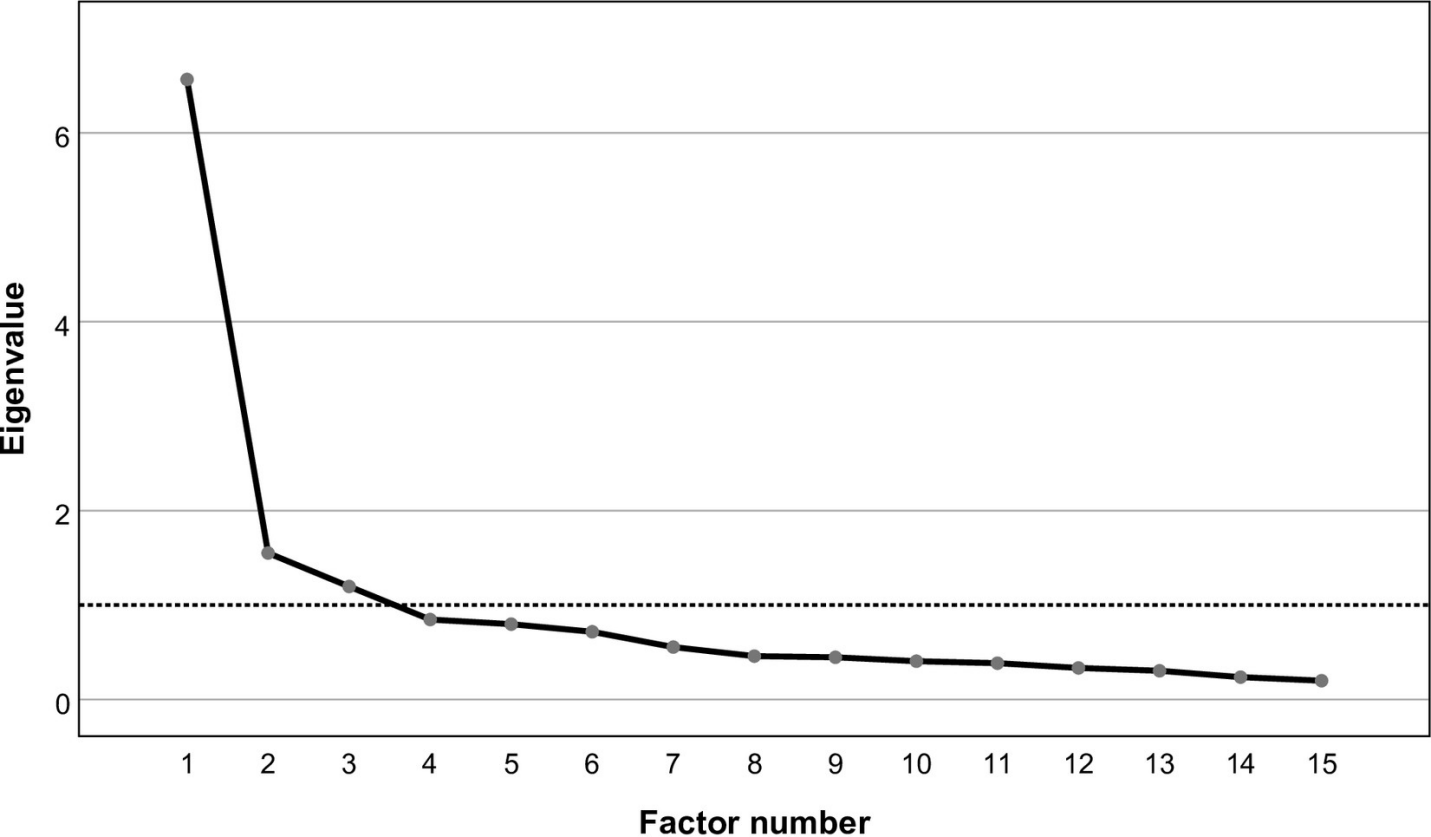
Scree plot showing the variance explained by each factors to extract. It is recommended to extract factors with a factorial load greater than 1 that are located above the "elbow" of the graph, that is, the factors above the dashed line.

In order to decide how many factors should be retained, the Kaiser-Guttman rule (retain factors with Eigenvalues higher than 1) and a visual inspection of the Scree plot (Fig 2) was used. This analysis revealed that 3 factors should be retained.

In EFA, the eigenvalues and the percentage of variance explained by means of each of these factors were determined. The total of the factors extracted explained 62.1% of the total variation of the questionnaire. The first factor explained 43.8% of the total variation, the second, 10.3% and the third, 8%. Table 1 presents the factorial weights of each item for each of the three factors. A cut-off point of 0.4 was used for the interpretation of the factorial weights of the items.

**Table 1:** Factor loadings after rotation.

Items	Factor 1	Factor 2	Factor 3
1. Eye tiredness related to near activities	0.952		
2. Eye discomfort related to near activities	0.879		
3. Headaches related to near activities	0.792		
5. Concentration loss related to near activities	0.670		
4. Sleepiness related to near activities	0.610		
10. Eye pain related to near activities	0.592		
11. Eye soreness related to near activities	0.511		
12. Eye "pulling" feeling related to near activities	0.482		
7. Double vision related to near activities		0.817	
8. Text motion while reading		0.776	
13. Text defocusing in near activities		0.722	
15. Need to re-read the same line of words			0.857
9. Slow reading sensation			0.774
6. Trouble remembering reading text			0.695
14. Loss of orientation in text during reading			0.604

Table 2 presents the percentage of variance explained, naming and internal reliability by each factor. The internal consistency of each of the extracted factors, inferred through the Cronbach coefficient, reveals good reliability (α > 0.7) for all factors retained.

**Table 2:** Total variance explained, naming and internal reliability by extracted factors.

Parameter	Factor 1	Factor 2	Factor 3
Explained variance	43.8%	10.3%	8%
Extracted Factors Naming	Somatic Sensations	Impaired vision	Cognitive performance
Internal reliability—Chronbach’s alpha	0.893	0.765	0.762

Factor 1 included the items of the questionnaire regarding the sensation of pain, discomfort and ocular tiredness (Item 1, 2, 10, 11 and 12); headaches (Item 3), and drowsiness during reading and loss of concentration (Item 4 and 5). Given the characteristics of these variables, this factor was named "somatic sensations".

Factor 2 grouped items that questioned about diplopia (Item 7), text movement and intermittent blurring during near tasks (items 8 and 13). These situations represent the propensity for deficits in binocular vision, and are effectively symptoms that affect the quality of vision, reason why this factor was designated "impaired vision".

Factor 3 consisted of items that reported on the need for re-reading, difficulties in remembering what was read, and slow reading (Item 15, 6 and 9), as well as getting lost in the text while reading (Item 14). These symptoms represent potential problems associated with poor reading and were therefore referred to as "cognitive performance".

## Discussion

Exploratory factorial analysis is one of the statistical procedures mostly used in the development, evaluation and refinement of questionnaires, and has become a widely used statistical method in the field of psychology, education, health, biology and other areas. AFE is defined as a set of multivariate techniques that aims to find an underlying structure in a data matrix and determine the number and nature of the latent variables (factors or dimensions) that best represent a set of observed variables.

The instrument under study, the convergence insufficiency questionnaire (CISS) in Portuguese version, was a factorial data matrix (Bartlett's sphericity test: p <0.001) and revealed a good index for factorization (KMO = 0.89) [24,25]. The reduction of factors with the exploratory factorial analysis, using the principal component method, allowed the extraction of three latent dimensions, which explain 62.1% of the total variance.

In reports of the literature it is verified that the complaints of asthenopia are very disparate, which makes difficult to associate specific complaints to specific problems. However, the fragmentation of this group of symptoms is often found in two categories, either refractive and muscular [14], or internal and external symptoms [15]. The refractive asthenopia being associated with uncorrected refractive errors and the muscular asthenopia with changes in convergence and / or accommodation [14]. The internal symptoms being related to complaints of dry eye and ocular surface irritation, which represent symptoms primarily triggered by working conditions; and the external type related to symptoms of tension and ocular pain, symptoms primarily triggered by deficits in visual function [15].

For the instrument under study, it was not possible to associate the structure obtained with the terminology of “internal symptoms and external symptoms”, perhaps due to the basic structure of the questionnaire under analysis, where the characteristic symptom of dry eye is missing, being the nomenclature “refractive and muscular asthenopia” more appropriate for this instrument.

In previous studies, the CISS questionnaire was categorized into two sub-scales, one related to performance and another related to the eye [28], however, this division was not validated with any statistical model. Also Clark & Clark used the original questionnaire, creating distinct groups for 4 factors: fatigue, discomfort, impaired vision and cognitive performance [4], however, the statistical model that gave rise to this division is not explained.

According to the factorial structure obtained in the present study, for the CISS questionnaire, three factors were extracted. Factor 1, named "somatic sensations", joins the complaints that are mostly associated with accommodative effort [7,8,12] and refractive problems [9,11]. This factor may be representative of refractive asthenopia complaints, a term traditionally used to characterize the visual complaints associated with refraction [14] and includes the main variables that constitute the "fatigue" and "discomfort" dimensions proposed in Clark's work [4]. Factor 2, referred to as "impaired vision", comprises complaints related to difficulties in maintaining binocular fixation that guarantees a clear and single view. This factor may be representative of complaints of muscular asthenopia, which refer to visual complaints associated with problems of binocular vision [14] and represents the subscore with the same name, suggested in other works [4]. Factor 3, which was denominated "cognitive performance", includes symptoms related to memory, attention and comprehension, which are considered reading skills and this factor is similar to one of Clark's analysis factors with the same name. These types of symptoms are associated to disorders in reading, in the presence, or not, of ocular problems [4,6].

The analysis of this questionnaire according to the proposed factorial structure allows us to look at the third factor (cognitive performance) as a factor that must be analyzed separately and by relative comparison with the other factors. A higher score on this third factor than on others may reveal that the case is primarily due to learning and reading difficulties. This analyses may help to clarify the relationship between binocular changes, problems in reading ability and attention deficit disorders, a fact pointed out by other authors as being lacking of more research [1,6,20].

Several studies associate complaints of visual discomfort with near-vision tasks, however, it is difficult to associate a specific type of complaint with a specific problem. It can be speculated that complaints of visual discomfort, with a higher relative score in factor 1 (somatic sensations) than in the other factors, may be attributed mainly to eye strain during near tasks for prolonged periods which require greater visual effort. Environmental conditions, uncorrected refractive errors and accommodative problems are often reported as the major causes of these type of symptoms [2,8,9]. On the other hand, since the severity of these changes tends to produce more uncomfortable and intense symptoms, which may include complaints of binocular quality loss [8,11] it is possible that a high score in factor 1 and in factor 2 (somatic sensation and impaired vision) is associated with visual changes with greater severity.

In conclusion, the factor structure of the CISS survey allows to extract three dimensions, each of which has satisfactory internal consistency, so that they can be used separately, each with a subscore, instead of a global score.

The diversity of common complaints to refractive errors and alterations in binocular vision has made the questionnaire use a useful tool in clinical routine. It is therefore necessary to explore the application of this questionnaire, analyzing its latent dimensions, in order to identify patterns of relative variation between each of the factors, which help both in diagnosing the conditions and in defining the best strategies in order to reduce the symptoms. In addition, the third dimension of this questionnaire (cognitive performance) can be very helpful in assessing children with difficulties in reading to help distinguish if the visual complaints relate only to the mental and cognitive state or if there is also impaired visual function. This research will be complemented with an external validation, applying the CISS questionnaire and analysing the results in the three suggested dimensions to specific populations.

## Supporting information

S1 Dataset (XLSX)

S2 Dataset (XLSX)
